# The Role of Autophagy in the Mother-to-Child Transmission of Pregnant Women With a High Level of HBV DNA

**DOI:** 10.3389/fcimb.2022.850747

**Published:** 2022-04-22

**Authors:** Hong Gao, Ling Xu, Zihao Fan, Xiangying Zhang, Zhongping Duan, Feng Ren

**Affiliations:** ^1^ Beijing Youan Hospital/Beijing Institute of Hepatology, Capital Medical University, Beijing, China; ^2^ Department of Gastroenterology, The Second Hospital of Shanxi Medical University, Taiyuan, China

**Keywords:** vertical transmission of HBV, autophagy, mother-to-child transmission, placenta, hepatitis B

## Abstract

**Background:**

Mother-to-child transmission (MTCT) is the most common propagation mode of hepatitis B virus (HBV) transmission. Exploring the mechanisms of HBV MTCT is the key to protect infant from infection. In this study, we aim to clarify the important role of autophagy complicated in HBV MTCT.

**Methods:**

A total of 169 placental samples were collected in this study, includes 144 HBV positive pregnant women and 25 normal pregnant women. *In vitro*, JEG-3 cells were treated with serum contained different HBV viral loads. Electron microscope was used to observed the number of autophagosome. RT-qPCR and western blotting were used to measure the expression level of autophagy relative genes and proteins respectively. Immunofluorescence was used to analyzed the expression of LC-3 of the frozen section of placental tissue.

**Results:**

According to the number of autophagosomes and the expression level of autophagic genes mRNA and protein, autophagy was increased in HBV maternal placenta. Among the control, low viral load, medium viral load and high viral load groups, autophagy was significantly up-regulated with the increase of HBV viral loads. Also, autophagy was increased in the HBeAg positive pregnant women compared with their HBeAg negative counterparts. Also, autophagy in infant-infected group was up-regulated compared with infant-uninfected group. *In vitro*, choriocarcinoma JEG-3 cells were treated with the different HBV viral loads or different time incubation, the mRNA and protein of autophagy related genes was maximum expression in the medium viral load or treatment in a short period, but decreased in the high viral load treatment or with long-term HBV exposure.

**Conclusion:**

Our study determines the high levels of viremia could be the cause of both increase autophagy activities and MTCT. Autophagy was significantly up-regulated in pregnant women with high viral load or HBeAg positive, which plays an important part in the HBV MTCT.

## Introduction

Hepatitis B virus (HBV) infection has become a key threat to human health. In China, approximately 5.2% of childbearing women are positive for hepatitis B surface antigen (HBsAg), and 28.78% are positive for hepatitis B e antigen (HBeAg) among HBsAg positive women (Zhang et al., 2017). Mother-to-child transmission (MTCT) is the common modes of HBV transmission (Chinese Society of Hepatology, Chinese Medical Association, 2017). Although long-term antiviral therapy and passive-active immunoprophylaxis are available, there are still a few newborns who acquire HBV infection (Yi et al., 2016).

There are three routes for MTCT: intrauterine infection, infection after birth and delivery infection (Pawlowska et al., 2016). A growing number of studies have verified that MTCT is more likely to occur in mothers with a high viral load (ranging from 10^5^-10^8^ IU/mL) or HBeAg positivity (Wen et al., 2013; Zhang et al., 2014; Liu et al., 2015). Reducing HBV DNA levels below 10^6^ IU/mL reduces the risk by approximately 30% (Yu et al., 2014). According to evidence from the European Association for the Study of Liver (EASL), mothers with an HBV DNA level >10^6^-10^7^ IU/ml require antiviral therapy to reduce the risk of MTCT (European Association for The Study of The Liver, 2012; Pan et al., 2016). Additionally, the Chinese Guidelines for the Prevention and Treatment of Chronic Hepatitis B (version 2019) determined that mothers with an HBV DNA level >2×10^5^ IU/ml require antiviral therapy to prevent MTCT (Chinese Society of Infectious Diseases, Chinese Medical Association; Chinese Society of Hepatology, Chinese Medical Association, 2019).

Some studies have made progress on the mechanism of HBV vertical transmission. Hepatitis B immunoglobulin (HBIG) administration can form an immune barrier between the mother and fetus to prevent HBV transmission (Liu et al., 2015). Peripheral blood mononuclear cells (PBMCs) are the HBV extrahepatic replication site and contribute to HBV intrauterine infection (Shi et al., 2017). Some researchers have concluded that germline cell transmission may play an important role in vertical transmission (Mavilia and Wu, 2017). However, the precise molecular mechanism of MTCT has not been clarified, especially in pregnant women with a high level of HBV DNA or with serum HBeAg positivity. Autophagy is an evolutionarily conserved degradation process that is involved in immune responses to various pathogens. Some studies have shown that autophagy can support the replication of many viruses, such as coronaviruses, flaviviruses, and hepatitis C virus (HCV) (Chan and Ou, 2017; Chiramel and Best, 2018; Miller et al., 2020). Thus, autophagy in placental cells may play an important role in the vertical transmission of HBV.

Previous studies have shown that induction of trophoblast autophagy *in vitro* significantly improves antiviral activity, which plays a critical role in HBV MTCT (Glick et al., 2010). High HBV loads and HBeAg positivity are the two major factors that result in failure of block transmission from mother to child. In this study, we aim at analyzing the activities of autophagy in pregnant women with hepatitis B according to two factors: the HBV viral load and HBeAg positivity status. We hypothesis that high levels of viremia could be the cause of both increase autophagy activities and MTCT.

## Materials And Methods

### Ethical Approval

The protocol of the current investigation was approved by the medical ethics committee of Beijing Youan Hospital, Beijing, China. Informed consent was obtained from the patients. The study was conducted in accordance with the ethical principles of the Declaration of Helsinki established in 1975 and revised in 1983. All the subjects were accepted clinical management according to management algorithm for interrupting mother-to-child transmission of hepatitis B (2017) with informed consent.

### Study Cohort and Patient Characteristics

A retrospectively research was conducted included a total of 169 pregnant women (Normal:25 cases and 144 cases with chronic hepatitis B) at Beijing You An Hospital Capital Medical University from January to December 2016. Demographic data and prenatal serological test results for the patient cohort were obtained from medical records. Among these women, we have excluded patients who were infected with hepatitis A virus (HAV), HCV, hepatitis D virus (HDV), hepatitis E virus (HEV), Epstein-Barr virus (EBV), cytomegalovirus (CMV), or human immunodeficiency virus (HIV) or had other diseases from this study. As standard of care, HBV vaccination (10 μg) within 12 hours of birth and the administration of 100 IU HBIG, were provided to all newborns (regardless of whether the newborn was infected or not).

Among the 144 pregnant women with chronic hepatitis B, 87 of them were divided into 3 groups according to prenatal HBV DNA level quantification: 35 patients (viral load <10^3^ IU/mL) were included in the low viral load group, 34 patients were included in the medium viral load group (10^3^ IU/mL ≤viral load<10^6^ IU/mL), and 18 patients were included in the high viral load group (viral load ≥10^6^ IU/mL). 57 of them were divided into two groups according to their differential expression of HBeAg: 27 patients were included in the HBeAg-positive group, and 30 patients were included in the HBeAg-negative group. In addition, among the 144 pregnant women with HBV infection, two of them were mother-infant immunoprophylaxis failure and 142 of mothers’ immunoprophylaxis successful.

Placentas were collected from the enrolled pregnant women with singleton pregnancies who delivered at term (the boundary of the placenta and fractions of calcification, bleeding and necrosis were avoided). The placentas were reserved after delivery for 10 min and then washed three times with distilled water to remove the maternal blood. Placental cotyledons were dissected in the middle zone, containing all placental cell layers, and measured approximately 1.5* 2* 3 cm. Then, the samples were placed into aseptic cryovials and quickly preserved in liquid nitrogen. They were saved in a freezer at -80°C until analysis.

### HBV DNA Detection System

Detection of HBV DNA was performed on an Abbott M2000 RT real-time instrument. 200 μL of serum from enrolled subjects was used for the nucleic acid extraction, the operation conducted according to the manufacturer’s instructions.

### Reverse Transcription-Quantitative Polymerase Chain Reaction (RT-qPCR) Analysis

Total RNA was isolated from placental tissues using TRIzol reagent (Invitrogen Life Technologies, Carlsbad, CA) and then reverse transcribed into cDNA with the SuperScript™ III First-Strand Synthesis System according to the instructions. The PCR system for each well included 4 µl cDNA, 0.8 µl primer, 5.2 µl diethylpyrocarbonate water and 10 µl SYBR Green mixture, which was then used for amplification on a quantitative PCR instrument (ABI Prism 7500; Applied Biosystems Inc., Waltham, MA, USA). The thermal cycling conditions were 50°C for 2 min and 95°C for 5 min, followed by 50 cycles of 95°C for 15 s and 60°C for 30 s. Hypoxanthine phosphoribosyl transferase (HPRT) was selected as an endogenous control. Target mRNA levels were analyzed by the cycle threshold (Ct) method and were normalized to HPRT levels.

### Western Blot Analysis

Protein was extracted from placental tissue in RIPA buffer with phosphatase and protease inhibitors. A total of 40 µg protein was separated by 12% polyacrylamide gel electrophoresis and then transferred to a PVDF membrane (Bio-Rad, Hercules, CA). Antibodies against target proteins (1:1,000; Cell Signaling Technology Inc., Santa Cruz, CA) were incubated with membranes at 4°C overnight. After washing with TBST buffer 3 times, the membranes were further incubated with a horseradish peroxidase-conjugated secondary antibody (1:2,000; Cell Signaling Technology Inc., Santa Cruz, CA) for 1 hour at room temperature. A commercial enhanced chemiluminescence kit (Thermo Fisher Scientific, Rockford, IL) was used for target protein visualization.

### Immunofluorescence

The sections were fixed with paraformaldehyde for 10 min. Then, they were washed with precooled 0.01 M phosphate-buffered saline (PBS), pH 7.2, three times for 5 min each time. The sections were blocked with 1% Triton X-100 for 15 min. Then, they were blocked with 10% goat serum for 1 hour at 37°C. The sections were incubated at 4°C overnight with a mouse monoclonal antibody. Then, they were washed with PBS three times for 5 min each time. We incubated the sections with fluorochrome-labeled secondary antibodies for 1 hour at room temperature. Then, 20 μL DAPI was added to each of the slides to visualize nuclei. Immunofluorescence images (40x10) were acquired using an Eclipse E800 fluorescence microscope.

### Cell Culture and Treatment

HBV carrier samples (normal liver function) with different levels of HBV DNA (10^2^, 10^4^, 10^6^, and 10^8^ IU/ml) and healthy volunteer samples were collected. Serum samples were filtered through a 0.22-μm ion device (Costar Co., Ltd.) to eliminate any bacteria. Then, the samples were treated in a water bath at 56°C for 30 min to inactivate complement components.

JEG-3 cells were grown in DMEM/F12 (1:1) supplemented with 10% fetal bovine serum (FBS) and antibiotics at 37°C and 5% CO_2_. Cells were seeded at 2×10^5^ cells/mL and cultured overnight, after which the medium was changed. For different viral load treatments, once cells reached 50-60% confluence, serum samples from healthy volunteers and those from HBV carriers with different viral loads (10^2^, 10^4^, 10^6^, and 10^8^ IU/ml) were added and incubated for 48 hours. For different treatment times, serum from HBV carriers (2.4×10^8^) was incubated for 3, 6, 12 or 24 hours. We washed the treated cells three times with PBS and collected them. We repeated the experiments at least three times.

### Statistical Analysis

Values are presented as the mean ± SD. Statistical analyses were performed using IBM SPSS Statistics for Windows, version 13.0 (IBM Corp., Armonk, NY, USA). One-way analyses of variance were used to compare measured parameters among multiple groups (n>=3). A t-test was used for samples with homogeneous variance, and the Games-Howell test was used for samples with heterogeneous variance. P values are indicated by labels, and a p-value less than 0.05 was considered significant.

## Results

### Clinical Data

The clinical characteristics and details of the participants enrolled in the study are summarized in **Tables 1** and **2**. **Table 1** presents the demographic and clinical characteristics of the different HBV load groups. The levels of alanine aminotransferase (ALT), aspartate aminotransferase (AST), alkaline phosphatase (ALP) and total bilirubin (Tbil) showed obvious differences among the low, medium, and high viral load groups. And **Table 2** present the characteristics of different HBeAg status groups. The analyzed clinical parameters were the same as **Table 1**; pregnant women who were HBeAg positive showed worse liver function than those who were negative. In addition, we found HBV infection in placenta of all HBV pregnant women using fluorescence staining with HBsAg (**Figure S1**).

**Table 1:** Clinical data of different HBV viral load groups.

	Normal group(n=25)	Low virus load(HBV loads ≤ 10^3^, n=35)	medium viral load (10^3^<HBV loads ≤ 10^6^, n=34)	High viral load(HBV loads>10^6^, n=18)	P
Maternal age(y)	30.4±5.9	31.2±3.9	30.2±4.3	30.4±5.0	0.75
Gestational weeks (w)	39.0±1.2	38.9±1.3	39.0±1.3	38.8±0.7	0.973
Neonatal birth weight(g)	3357.1±418.8	3304.6±576.	3340.1±392.7	3332.5±435.8	0.975
Alanine aminotransferase (ALT, U/L)	18.1±2.2	20.1±8.7	32.8±11.1	40.9±15.2	0.013
Aspartate aminotransferase (AST, U/L)	19.5±2.8	23.6±9.9	34.6±12.7	43.6±13.6	0.022
Alkaline phosphatase (ALP, U/L)	110.38±50.1	125.1±63.7	136.9±53.5	136.3±22.7	0.237
Total bilirubin (TBil,µmol/L)	8.2±3.1	9.6±5.9	10.7±6.8	9.9±6.0	0.331
Direct bilirubin (DBil, µmol/L)	3.2±1.3	3.6±2.6	3.8±3.5	3.0±1.5	0.77
HBV DNA (log_10_ IU/mL)	–	2.47±0.7	5.53±2.3	7.87±3.65	–

**Table 2:** Clinical data of different HBeAg expression groups.

	Normal group (n=25)	HBeAg positive (n=30)	HBeAg negative (n=27)	P
Maternal age(y)	30.4±5.9	30.92±3.91	29.94±4.46	0.213
Gestational weeks (w)	39.0±1.2	38.7±1.3	39.2±1.2	0.249
Neonatal birth weight(g)	3357.1±418.8	3264.4±544.8	3320.2±427.3	0.538
Alanine aminotransferase (ALT, U/L)	18.1±2.2	18.83±18.92	24.06±20.36	0.158
Aspartate aminotransferase (AST, U/L)	19.5±2.8	24.33±13.85	30.10±13.76	0.027
Alkaline phosphatase (ALP, U/L)	110.38±50.1	131.88±56.56	136.06±55.26	0.689
Total bilirubin (TBil,µmol/L)	8.2±3.1	11.09±7.34	9.12±4.15	0.089
Direct bilirubin (DBil, µmol/L)	3.2±1.3	3.5±1.4	3.8±2.9	0.127
HBV DNA (log_10_ IU/mL)	–	5.86±2.5	4.67±3.5	0.142

### Differential Expression of Autophagy Markers Between the Placenta of Normal and HBV-Positive Pregnant Women

**Figure 1:**
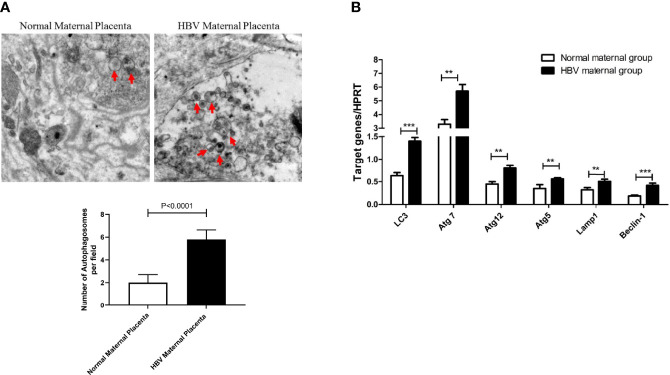
Differential expression of autophagy markers between the placenta of normal and HBV-positive pregnant women. (***p<0.001, **p<0.01). **(A)** The number of autophagosomes was detected by electron microscopy, the autophagosomes were indicated by red arrows and quantified by randomly selecting and screening 100 fields for two samples in the normal maternal group and two samples in the HBV maternal group. The representative images from the normal maternal group and HBV maternal group are shown. **(B)** mRNA expression of LC3, ATG7, ATG12, ATG5, Lamp1, Beclin-1 was measured by qPCR between the normal maternal group (25 cases) and HBV maternal group (144 cases). Data are shown as mean ± SD of at least three independent experiments.

First, with an electron microscope, we detected autophagosome formation in the placental tissues of normal and HBV-infected pregnant women. We found that the number of autophagosomes in HBV maternal placental tissue was increased significantly compared with that in normal maternal placental tissue (**Figure 1A**). Furthermore, the expression levels of multiple genes involved in autophagy were measured, and the results showed that the mRNA levels of LC3, Atg5, Atg7, Atg12, Lamp1, and Beclin-1 were all upregulated in the HBV-positive maternal placenta compared with the normal maternal placenta (**Figure 1B**). Together, the results indicate that autophagy is obviously activated in the placenta of pregnant women with HBV infection, which indicates that autophagy plays an important role in the vertical transmission of HBV.

### Expression Differences in Autophagy Markers in the Placenta of Pregnant Women With Different HBV Loads

**Figure 2:**
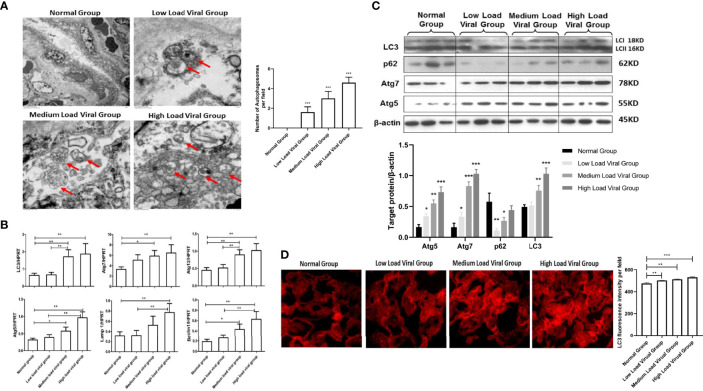
Expression differences in autophagy markers in the placenta of pregnant women with different HBV loads. (***p<0.001, **p<0.01, *p<0.05). **(A)** The number of autophagosomes was detected by electron microscopy, the autophagosomes were indicated by red arrows and quantified by randomly selecting and screening 100 fields for two samples in the normal group, low viral load group, medium viral load group, and high viral load group, respectively. The representative images from each group are shown. **(B)** mRNA expression of LC3, ATG7, ATG12, ATG5, Lamp1, Beclin-1 was measured by qPCR among low viral load group (35 cases), medium viral load group (34 cases), high viral load group (18 cases), and normal group (25 cases). Data are shown as mean ± SD of at least three independent experiments. **(C)** Protein expression of LC3, p62, ATG7, ATG5, and β-actin was measured by western blot among low viral load group (35 cases), medium viral load group (34 cases), high viral load group (18 cases), and normal group (25 cases). The results were repeated at least three times. The representative image from placental tissue of normal group and low, medium, high viral load groups are shown. **(D)** The protein levels of LC3 were measured with immunofluorescent assays in placental tissue of normal group, low, medium, high viral load groups (200×). The quantitative analysis of LC3 fluorescence intensity per field was examined by Image J.

Recent studies have shown that the HBV load is closely related to the failure to block mother-to-child transmission. We thought that the upregulation of autophagy marker expression in the placenta of HBV-positive pregnant women might be related to the HBV load. First, we detected the number of autophagosomes in the low, medium, and high viral load groups using electron microscopy. We found that the number of autophagosomes gradually increased as the HBV load in pregnant women increased (**Figure 2A**). Furthermore, we tested the expression levels of autophagy-related genes and proteins in the different HBV load groups. With increasing viral load, the mRNA levels of LC3, Atg5, Atg7, Atg12, Lamp1, and Beclin-1 were upregulated in HBV-positive pregnant women (**Figure 2B**). Similarly, we analyzed the protein levels of LC3, p62, Atg7, and Atg5 in the different HBV load groups, which showed the same expression profile as the mRNA levels of the autophagy-related genes (**Figure 2C**). In addition, we used fluorescence staining to detect the expression of LC3 in placentas with different viral loads. We observed that LC3 expression was upregulated with increasing HBV load in pregnant women (**Figure 2D**). Overall, the expression levels of autophagy markers are gradually increased with the HBV load in pregnant women with different viral loads.

### Expression Differences in Autophagy Markers in the Placenta of HBeAg-Positive or HBeAg-Negative Pregnant Women

**Figure 3:**
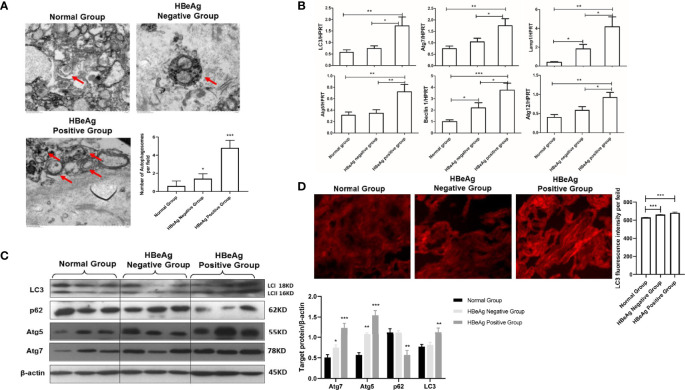
Expression differences in autophagy markers in the placenta of HBeAg-positive or HBeAg-negative pregnant women. (***p<0.001, **p<0.01, *p<0.05). **(A)** The number of autophagosomes was detected by electron microscopy, the autophagosomes were indicated by red arrows and quantified by randomly selecting and screening 100 fields for two samples in the normal group, HBeAg-positive group, and HBeAg-negative group, respectively. The representative images from each group are shown. **(B)** mRNA expression of LC3, ATG7, ATG12, ATG5, Lamp1, Beclin-1 was measured by qPCR among the normal group (25 cases), HBeAg-positive group (27 cases), and HBeAg-negative group (30 cases). Data are shown as mean ± SD of at least three independent experiments. **(C)** Protein expression of LC3, p62, ATG7, ATG5 and β-actin was measured by western blot among the normal group (25 cases), HBeAg-positive group (27 cases), and HBeAg-negative group (30 cases). The results were repeated at least three times. A representative image from placental tissue of normal group, HBeAg-positive group, and HBeAg-negative group is shown. **(D)** The protein levels of LC3 were measured with immunofluorescent assays in placental tissue of normal group, HBeAg-positive group, and HBeAg-negative group (200×). The quantitative analysis of LC3 fluorescence intensity per field was examined by Image J.

Some studies have shown that HBeAg positivity plays an important role in HBV vertical transmission. In this study, we measured the number of autophagosomes formed in placental tissues from HBeAg-positive and HBeAg-negative pregnant women with electron microscopy. We observed that the number of autophagosomes in the placenta of HBeAg-positive pregnant women was significantly higher than that in the placenta of HBeAg-negative pregnant women (**Figure 3A**). Furthermore, we evaluated the expression levels of several autophagy-related genes, including LC3, Atg5, Atg7, Atg12, Lamp1 and Beclin-1, in the placental tissue of HBeAg-positive and HBeAg-negative pregnant women. The results showed that the expression of autophagy-related genes was significantly increased in the HBeAg-positive group compared with the HBeAg-negative and normal groups (**Figure 3B**). Moreover, we analyzed the protein levels of LC3, Atg5, Atg7, and p62, which were upregulated in the HBeAg-positive group (**Figure 3C**). These data indicate that autophagy is activated in HBeAg-positive pregnant women. Likewise, LC3 fluorescence staining results showed that the expression of LC3 in the placenta of HBeAg-positive pregnant women was significantly higher than that in the placenta of HBeAg-negative pregnant women and normal controls (**Figure 3D**). Together, our data show that the number of autophagosomes increases and autophagy is significantly activated in the placenta of HBeAg-positive pregnant women.

### Expression Differences in Autophagy Markers Between the Mother-Infant Immunoprophylaxis Failure Group and Mother-Infant Immunoprophylaxis Group

**Figure 4:**
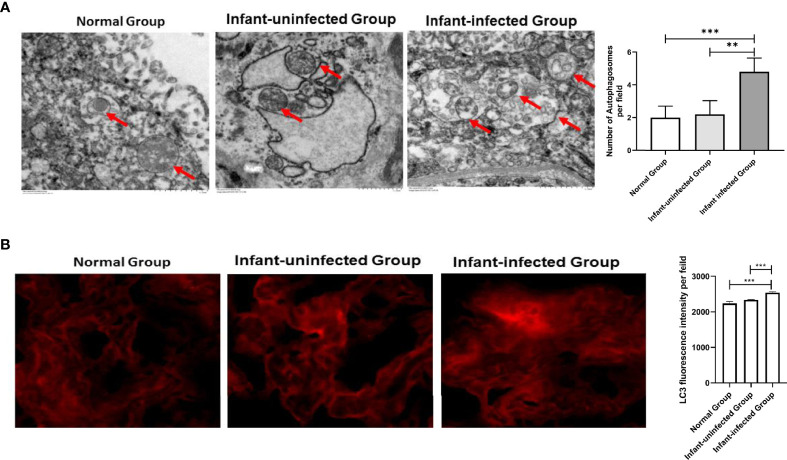
Expression differences in autophagy markers between the mother-infant immunoprophylaxis failure group and mother-infant immunoprophylaxis group (***p<0.001, **p<0.01). **(A)** The number of autophagosomes was detected by electron microscopy, the autophagosomes were indicated by red arrows and quantified by randomly selecting and screening 100 fields for two samples in the infants-uninfected group and two samples in the infants-infected group. **(B)** The protein levels of LC3 were measured with immunofluorescent assays in placental tissue of normal group, infant-uninfected group, and infant-infected group (200×). The quantitative analysis of LC3 fluorescence intensity per field was examined by Image J.

To better define the critical role of autophagy in mother-infant immunoprophylaxis failure, we explored the expression differences in autophagy markers between the infected infant group and the uninfected infant group. There are only two mothers experience immunoprophylaxis failure, which characteristics of serological and virological markers are presented in supplement **Table 1**. First, we found that the number of autophagosomes in the placenta of mothers in the infected infant group was significantly higher than that in the placenta of mothers in the uninfected infant group by electron microscopy (**Figure 4A**). Next, LC3 fluorescence staining results indicated that the expression of LC3 was significantly upregulated in the infected infant group compared with the uninfected infant group and normal infant group (**Figure 4B**). Taken together, these data show that autophagy is overactivated in the context of failure to block mother-to-child HBV transmission.

### Effects of Serum Containing HBV on Autophagy in JEG-3 Cells

**Figure 5:**
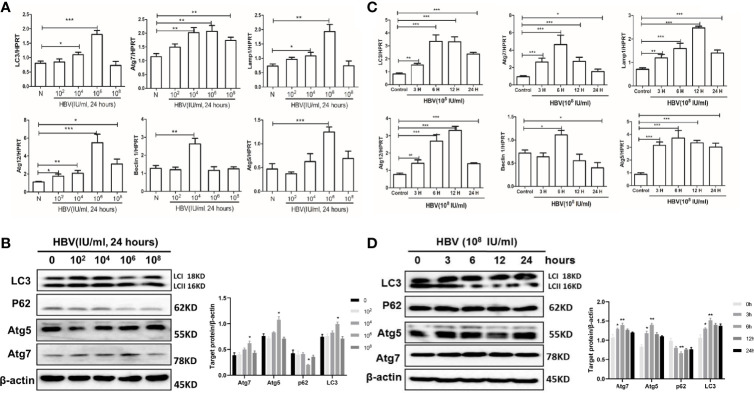
Effects of serum containing HBV on autophagy in JEG-3 cells. JEG-3 cells were treated with the medium containing 10% HBV serum. For different viral loads stimulation, serum samples from healthy volunteers and HBV carriers with different viral loads (10^2^, 10^4^, 10^6^, and 10^8^) were added and incubated for 48 hours; for different time periods of stimulation, serum from HBV carriers (2.4×10^8^) was incubated with cells for 3, 6, 12 or 24 hours. (***p<0.001, **p<0.01, *p<0.05). **(A)** mRNA expression of LC3, ATG7, ATG12, ATG5, Lamp1, Beclin-1 was measured by qPCR after stimulation by different viral load serum. Data are shown as mean ± SD of at least three independent experiments. **(B)** Protein levels of LC3, p62, ATG5, ATG7 were measured by western blot after stimulation by different viral load serum. The results were repeated by at least three independent experiments. **(C)** mRNA expression of LC3, ATG7, ATG12, ATG5, Lamp1, Beclin-1 was measured by qPCR after stimulation with HBV serum by different time periods. Data are shown as mean ± SD of at least three independent experiments. **(D)** Protein levels of LC3, p62, ATG5, ATG7 was measured by western blot after stimulation with HBV serum by different time periods. The results were repeated by at least three independent experiments.

To further explore the mechanism of HBV-induced autophagy in placental cells in the context of vertical transmission, we performed *in vitro* experiments with placental cells. First, cells were treated with serum samples containing different viral loads for 24 hours, and then we examined the expression of the autophagy-related genes LC3, Atg5, Atg7, Atg12, LAMP1 and Beclin-1. Strikingly, we observed the upregulation of LC3, Atg5, Atg7, Atg12, and LAMP1 expression when the HBV load was 10^6^ copies/ml and the upregulation of Beclin-1 expression when the HBV load was 10^4^ copies/ml (**Figure 5A**). Additionally, we analyzed the protein levels of LC3, Atg5, Atg7 and p62, which indicated that autophagy was activated by treatment with serum containing 10^6^ copies/ml HBV (**Figure 5B**). *In vitro*, autophagy was upregulated by treatment with serum containing 10^6^ copies/ml HBV; however, autophagy was downregulated by treatment with serum containing 10^8^ copies/ml HBV.

Then, we exposed JEG-3 cells to serum containing 10^8^ copies/ml HBV for different time periods. We measured the expression levels of autophagy-related genes after treatment with virus-containing serum for the different time periods. We observed that LC3, Atg7, Beclin-1 and Atg5 expression was highest at 6 hours, and Lamp1 and Atg12 expression was highest at 12 hours (**Figure 5C**). Simultaneously, we measured the protein levels of LC3, p62, Atg5, and Atg7 after treatment with serum containing HBV for the different time periods (**Figure 5D**). All these data show that autophagy is activated after 6 hours of exposure to serum containing HBV; however, longer stimulation downregulates autophagy in cells.

## Discussion

The activation of autophagy in placental cells plays an important role in the mechanism of HBV MTCT (Jackson, 2013). According to previous studies, the HBV viral load and HBeAg status are considered the two primary factors for the vertical transmission of HBV (Wang et al., 2019). In view of this discovery, our study first showed that there was significant autophagy activation in the placental tissue of HBV pregnant women compared with that of normal pregnant women. Then, we found that autophagy activation in the placental tissue of pregnant women with HBV was obviously increased in the mothers with a high HBV load and those who were HBeAg positive. All the subjects were accepted clinical management according to *management algorithm for interrupting mother-to-child transmission of hepatitis B (2017)* with informed consent, although studies have reported the anti-HBV therapy might repress autophagy (Lin et al., 2020), the baseline of enrolled pregnants in our study is consistent. *In vitro*, we incubated JEG-3 cells with serum containing different HBV loads or incubated these cells with HBV-positive serum for different time periods to further explore the relationship between HBV infection and autophagy. Interestingly, with increasing HBV load or stimulation time, autophagy was first activated and then decreased. In our study, we demonstrated the important role of autophagy in HBV intrauterine infection, especially in pregnant women with a high HBV load or HBeAg positivity.

The placenta is a physical barrier between the mother and the fetus that is closely related to the mother-to-child transmission of many pathogens, including viruses (Cheung et al., 2019). In recent years, with the administration of HBIG and the hepatitis B vaccine, most MTCT of HBV has been effectively controlled (Ma et al., 2014). However, MTCT of HBV in pregnant women with a high HBV load is still an unresolved problem, especially transmission by intrauterine infection. HBV replication in PBMCs, the extrahepatic reservoir of HBV, is an important mechanism involved in MTCT (Jackson, 2015). Previous reports of transplacental leakage of maternal blood causing MTCT have been confirmed (Xu et al., 2002). HBV covalently closed circular DNA (cccDNA) is found in the ovaries and has a close relationship with the vertical transmission of HBV (Yu et al., 2012). Recently, published studies have shown that HBV infection can induce autophagy in host cells to promote viral replication and pathogenesis (Xie et al., 2018). In this study, we proposed a critical role for placental autophagy in HBV MTCT, which was found to be activated in pregnant women with a high HBV load, and HBeAg positivity in pregnant women might be a cause of the failure to block HBV MTCT.

Because of the unique role of autophagy in removing cytoplasmic components in a lysosome-dependent manner, autophagy also plays an important role in the elimination of intracellular pathogens. Autophagy can activate the innate immune response by regulating the production of inflammatory cytokines, such as type I interferons and IL-1β, and controlling the activation of the NLRP3 and AIM inflammasomes and antigen processing and presentation (Lapaquette et al., 2015). Recently, studies have shown that HBV infection can induce autophagy in a manner dependent on the HBS and HBX proteins (Liu et al., 2019). Activation of autophagy promotes HBV DNA replication, viral particle envelopment and HBV release from host cells. HBX activates the autophagy signaling pathway by binding with phosphatidylinositol-3-kinase class 3 (PI3KC3) and death-associated protein kinase (DAPK) (Wang et al., 2019) or directly upregulating the expression of Beclin-1, which upregulates the initial stage of autophagy. Although autophagy was demonstrated to play a critical role in HBV infection, there are few studies reported autophagy implicated in HBV MTCT. We identified the characteristics of autophagy activation in the placenta of mothers with HBV infection in the present study.

Overall, positivity for HBeAg and a high viral load in mothers are the two most important risk factors related to the MTCT of HBV (Shih and Liu, 2017). Studies have shown that high maternal levels of HBV DNA increase the rate of intrauterine infection, which is the most likely cause of mother-to-child immunoprophylaxis failure (Zhang et al., 2014). Additionally, studies have shown that HBeAg-positive mothers may suppress fetal immune function by increasing the proportion of regulatory T cells (Tregs), which leads to an increased risk of intrauterine transmission (Hao et al., 2017). In our study, we further confirmed that autophagy was upregulated in mothers with a high viral load or HBeAg positivity, which might be the key mechanism in HBV transmission between mothers and infants. However, there are some limitations in our study. Firstly, our results that autophagy activities in the placenta increased in HBeAg^+^ or highly viremic mothers are inconsistent with the results of *in vitro* experiment, which was increased in medium viral load and decreased in high viral load. The difference was due to the microenvironment of the placenta in mothers is not absolutely the same as cell line *in vitro*, the former may be more complicated. Currently, there are no models that could completely simulate the placental environment. Therefore, experiments *in vitro* could partially simulate the situation of the placenta, and the results further explain the relationship between autophagy of placental cells and HBV viral load, but the more detailed mechanisms still need our further exploration. Secondly, the cases included in the mother-to-child HBV immunoprophylaxis failure group is limited (2 cases). It is difficult to collect samples of the infant-infected cases since the combination of passive and active immunization significantly reduces MTCT of HBV in newborns of HBsAg+ mothers.

In conclusion, our study showed that autophagy was characteristically activated in the placenta of HBV-infected mothers implying that autophagy may play an important role in MTCT of HBV. In pregnant women who were HBeAg positive or had a high viral load, autophagy was upregulated in placental tissue and promoted transmission from mother to fetus. In the immunoprophylaxis of mother-to-child HBV transmission in the clinic, autophagy may be the key target; thus, this study provides new insight to develop a more effective way to block MTCT of HBV.

## Data Availability Statement

The original contributions presented in the study are included in the article/**Supplementary Material**. Further inquiries can be directed to the corresponding authors.

## Ethics Statement

The studies involving human participants were reviewed and approved by the medical ethics committee of Beijing Youan Hospital, Beijing, China. The patients/participants provided their written informed consent to participate in this study.

## Author Contributions

FR and ZD designed the paper. HG and LX performed and analyzed experiments and wrote the paper. ZF and XZ analyzed data and reviewed the paper. FR designed, supervised, and analyzed experimental work and reviewed the paper. All authors contributed to the article and approved the submitted version.

## Funding

This study was supported by the National Natural Science Foundation of China (81770611, 82002243), the Demonstrating Application and Research of Clinical Diagnosis and Treatment Technology in Beijing (Z191100006619096 and Z191100006619097), Key Projects of the Beijing Municipal Education Commission’s Science and Technology Plan (KZ202010025035) and Beijing Municipal Administration of Hospitals Clinical Medicine Development of Special Funding Support (XMLX201830). Special key research project of capital health development scientific research (2020-1-1151, 2021-1G-2181).

## Conflict of Interest

The authors declare that the research was conducted in the absence of any commercial or financial relationships that could be construed as a potential conflict of interest.

## Publisher’s Note

All claims expressed in this article are solely those of the authors and do not necessarily represent those of their affiliated organizations, or those of the publisher, the editors and the reviewers. Any product that may be evaluated in this article, or claim that may be made by its manufacturer, is not guaranteed or endorsed by the publisher.
