# Supplementary material for: The Role of Autophagy in the Mother-to-Child Transmission of Pregnant Women With a High Level of HBV DNA
Source: Front Cell Infect Microbiol. 2022 Apr 22;12:850747. doi: 10.3389/fcimb.2022.850747 (PMC9072787; doi:10.3389/fcimb.2022.850747)
Supplement: Supplementary file 1 [file DataSheet_1.pdf]

Supplement materials

Supplement figure 1. The expression of HBsAg in infant-uninfected group and infant-infected group

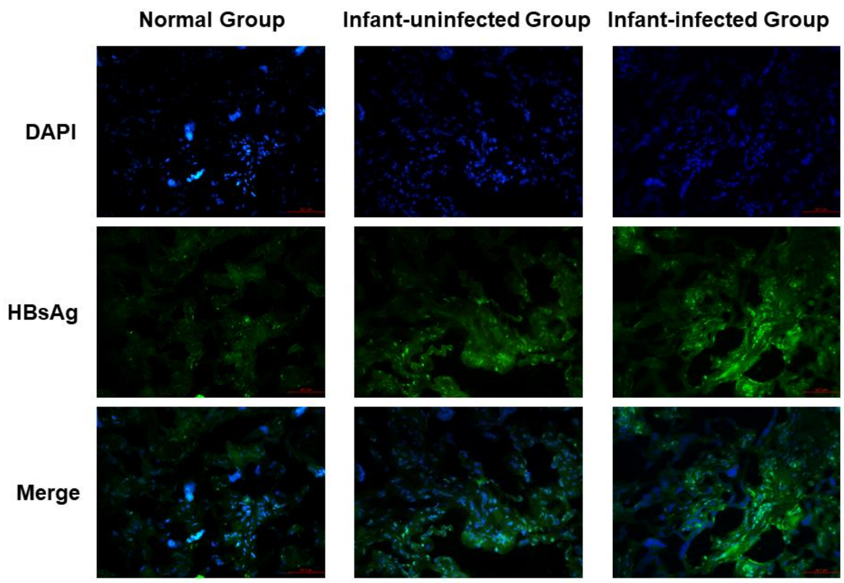

The protein levels of HBsAg were measured with immunofluorescent assays in placental tissue of normal group, infant-uninfected group, and infant-infected group (200×). The image was representing for each group.

Supplement Table 1. Clinical data of mother-infant immunoprophylaxis failure group and mother-infant immunoprophylaxis success group

	**mother-infant immunoprophylaxis failure group (n=2)**	**mother-infant immunoprophylaxis success group (n=142)**	**P**
Gestational weeks (w)	39.0	38.6±1.0	0.866
Neonatal birth weight(g)	3029.4	3531.09±827	0.509
Alanine aminotransferase (ALT, U/L)	27.8	17.8±5.5	0.035
Aspartate aminotransferase (AST,	22.5	20.9±7.8	0.031

U/L)			
Alkaline phosphatase (ALP, U/L)	321.5	223.0±48.7	0.049
Total bilirubin (TBil,μmol/L)	12.2	13.79±6.2	0.022
Direct bilirubin (DBil, μmol/L)	5.9	5.4±1.7	0.097
HBsAg (positive percentage)	100%	100%	>0.05
Anti-HBs (positive percentage)	0	87%	<0.001
HBeAg (positive percentage)	100%	59%	<0.001
Anti-HBe (positive percentage)	0	74%	<0.001
Anti-HBc (positive percentage)	100%	100%	>0.05
HBV DNA (log_{10} IU/mL)	8.22	3.42±0.3	0.042
